# First automatic size measurements for the separation of dwarf birch and tree birch pollen in MIS 6 to MIS 1 records from Northern Germany

**DOI:** 10.1002/ece3.11510

**Published:** 2024-06-14

**Authors:** Martin Theuerkauf, Elias Nehring, Alexander Gillert, Philipp Morten Bodien, Michael Hein, Brigitte Urban

**Affiliations:** ^1^ Institute of Ecology Leuphana University Lüneburg Lüneburg Germany; ^2^ Department of Biosystems Science and Engineering ETH Zürich Zürich Switzerland; ^3^ Fraunhofer Institute for Computer Graphics Research IGD Rostock Germany; ^4^ Institute for Botany and Landscape Ecology University of Greifswald Greifswald Germany

**Keywords:** automatic pollen recognition, convolutional neural networks, dwarf birch, Holocene, machine learning, middle and upper Pleistocene, TOFSI, tree birch

## Abstract

During past glacial periods, the land cover of Northern Eurasia and North America repeatedly shifted between open steppe tundra and boreal/temperate forest. Tracking these changes and estimating the coverage of open versus forested vegetation in past glacial and interglacial landscapes is notoriously difficult because the characteristic dwarf birches of the tundra and the tree birches of the boreal and temperate forests produce similar pollen grains that are difficult to distinguish in the pollen record. One objective approach to separating dwarf birch pollen from tree birch pollen is to use grain size statistics. However, the required grain size measurements are time‐consuming and, therefore, rarely produced. Here, we present an approach to automatic size measurement based on image recognition with convolutional neural networks and machine learning. It includes three main steps. First, the TOFSI algorithm is applied to detect and classify pollen, including birch pollen, in lake sediment samples. Second, a Resnet‐18 neural network is applied to select the birch pollen suitable for measurement. Third, semantic segmentation is applied to detect the outline and the area and mean width of each detected birch pollen grain. Test applications with two pollen records from Northern Germany, one covering the Lateglacial‐Early Holocene transition and the other covering the Mid to Late Pleistocene transition, show that the new technical approach is well suited to measure the area and mean width of birch pollen rapidly (>1000 per hour) and with high accuracy. Our new network‐based tool facilitates more regular size measurements of birch pollen. Expanded analysis of modern birch pollen will help to better understand size variations in birch pollen between birch species and in response to environmental factors as well as differential sample preparation. Analysis of fossil samples will allow better quantification of dwarf birch versus tree birch in past environments.

## Graphical abstract

Using image recognition with a series of convolutional networks and machine learning, we automatically measure the size of fossil birch pollen from sediment samples. The measurements are useful for determining the presence of dwarf birches versus tree birches in the past.

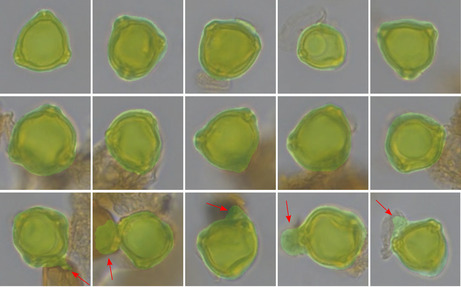

## 1 INTRODUCTION

Dwarf and tree birches are characteristic of different biomes, i.e., arctic‐alpine tundra versus boreal and temperate forests. Separating dwarf birch pollen from tree birch pollen is therefore a critical task in palynology, particularly for cold periods of the past, when in Europe and North America open (steppe)‐tundra existed farther south than today. Separating tree and dwarf birch pollen in the fossil record is difficult, however, because their pollen is so similar. Some authors suggest that separation is possible based on morphological features alone. Blackmore et al. (2003) point out that pollen grains of the European dwarf birch *Betula nana* have a more irregular outline of the ectoapertures, a smaller size of the vestibulum with less protruding apertures and a larger endo‐ than ectoaperture. In fossil pollen, however, these features are often difficult to detect, so that such morphological distinction is elsewhere considered unreliable (e.g., Birks, 1968). Alternatively, tree and dwarf birch pollen can be separated on the basis of their size (e.g., Jentys‐Szaferova 1928, Welten, 1944; Eneroth, 1951; Birks, 1968; Usinger, 1975; 1978; 1981; 1982; Gordon & Prentice, 1977; Kolstrup, 1982; Caseldine, 2001). Grain sizes are indeed different, but only for some taxa. While, for example, the pollen diameter of the tree birch *Betula tortuosa* is significantly larger than that of the dwarf birch *B. nana*, the diameter of *B. pubescens* pollen largely overlaps with both (Birks, 1968). Alternatively, the ratio of grain diameter to pore depth can be used to distinguish tree birch pollen from dwarf birch pollen, but again only for some taxa. This ratio separates pollen grains of *B. nana* and *B. pubescens*, but not those of *B. tortuosa* (Birks, 1968). For North America, the grain diameter of five tree and dwarf birch species do not differ significantly, and the ratio of grain diameter to pore depth only significantly separates between some pairs of species (Clegg et al., 2005). Therefore, also size measurements are not suited to unequivocally identify single grains as either tree or dwarf birch pollen.

The likely most robust way to separate dwarf birch pollen from tree birch pollen is to use grain size statistics from a large enough number of birch pollen (e.g., Usinger, 1978, 1981). The approach assumes that for each birch species, the pollen grain diameter is normally distributed, i.e. the grain diameter distribution is unimodal. In a mixed sample with pollen from two or more birch species, the grain size distribution would instead be different, in some cases bimodal or multimodal. Changes in grain size distributions along a pollen record, therefore, indicate changes in birch species composition. For each sample, the proportion of dwarf birch pollen and tree birch pollen can be approximated using numerical approaches (Prentice, 1981; Usinger, 1978, 1981). So far, applications of this method are rare, mainly because of the necessary, time‐consuming size measurements (e.g., Krüger & Damrath, 2020).

**FIGURE 1:**
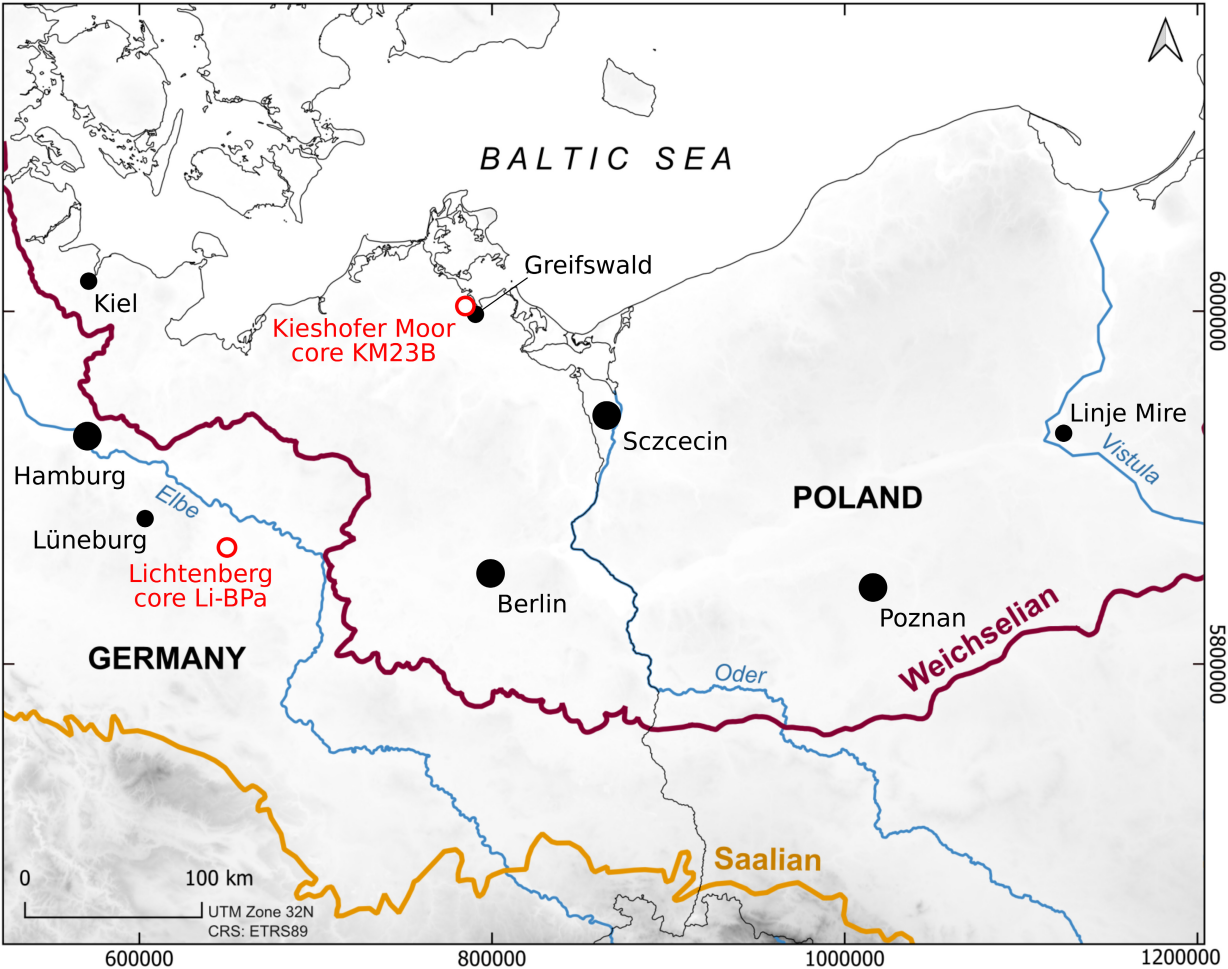
Map of the sample sites for modern birch pollen (black dots) and the location of the two fossil pollen records analysed in this study (red circles). The maximum glacier extents of the Weichselian and Saalian glacials are shown for reference. Created using QGIS 3.28.3 and Mapzen Global Terrain Data.

To overcome this limitation, we present a machine learning approach for the fast, automatic size measurement of birch pollen. The approach involves three main steps: first, the detection of birch pollen in microscope images of pollen samples; second, the selection of birch pollen suitable for measurement; and third, the actual size measurement. We present the approach and illustrate its application using modern reference material and fossil pollen samples from two sites in northern Germany, a Lateglacial to Early Holocene sequence from the Kieshofer Moor, Mecklenburg‐Vorpommern, and a Late Saalian to Early Weichselian sequence from the well‐known archaeological site Lichtenberg, Lower Saxony (Hein et al., 2021; Veil et al., 1994; Weiss et al., 2022), (Figure 1).

## 2 METHODS

### 2.1 Automatic size measurements

**FIGURE 2:**
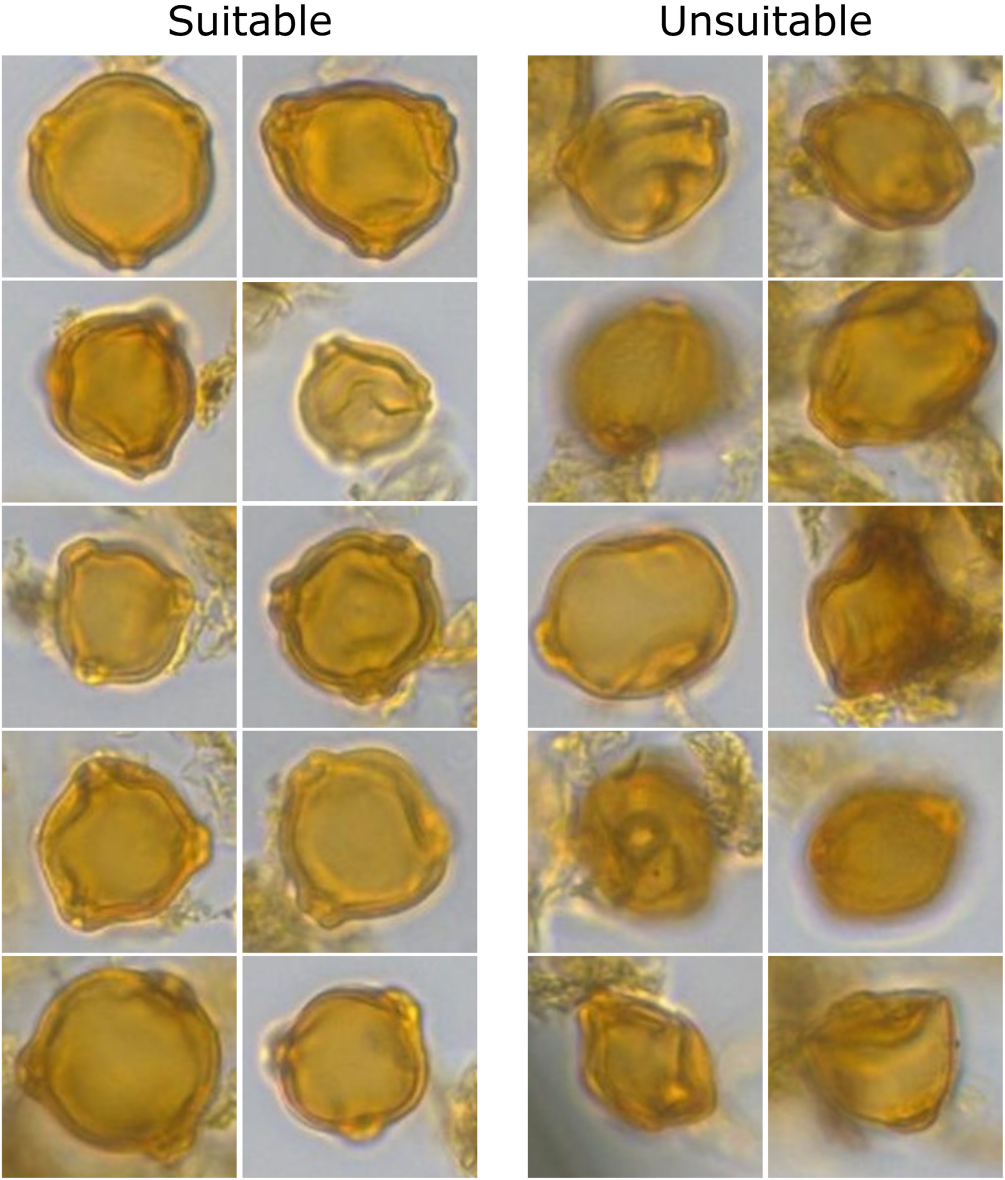
Pollen grains suitable/unsuitable for size measurement, automatically selected in the second step of our approach.

**FIGURE 3:**
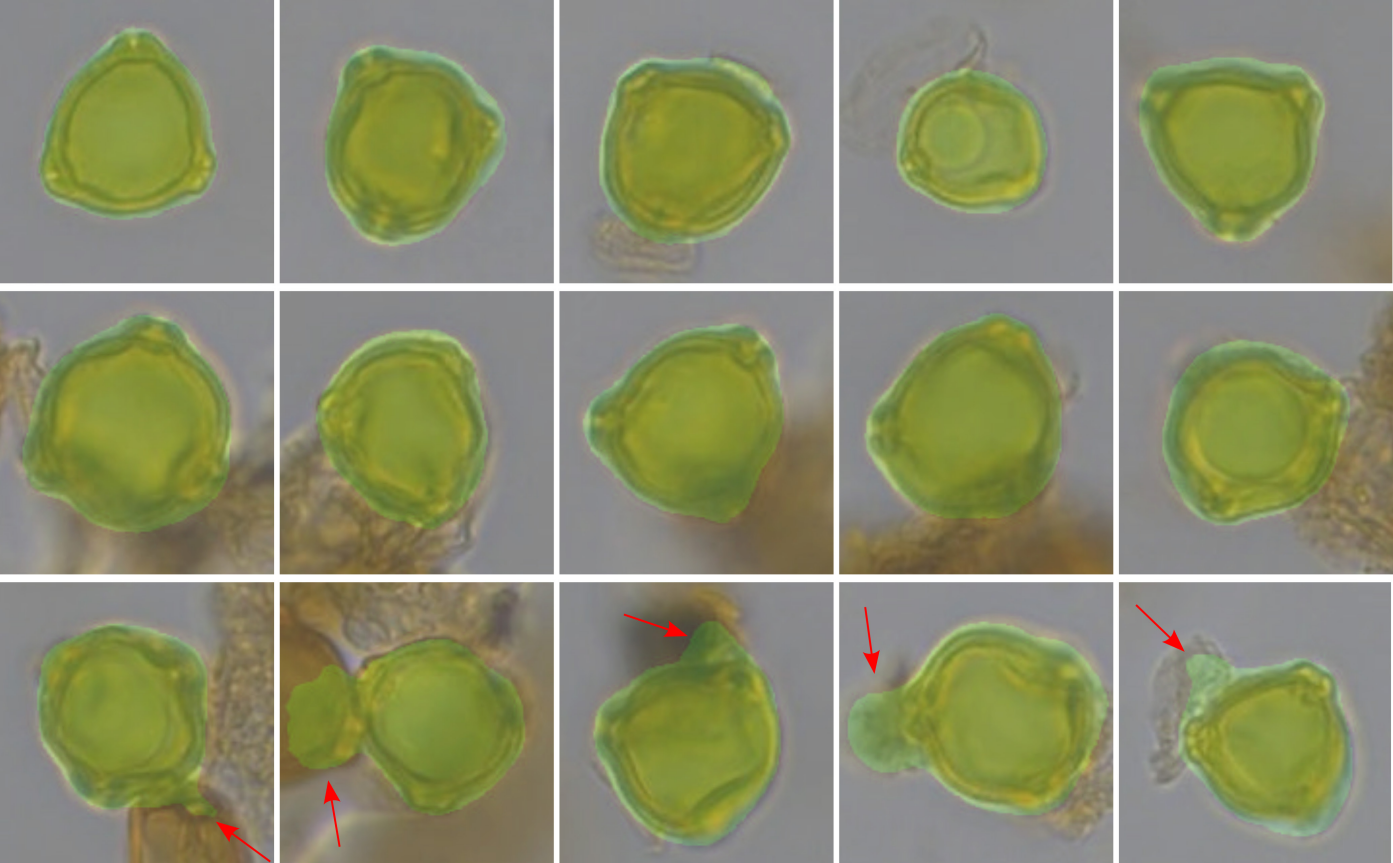
Application of step three of our approach, the automatic detection of the pollen grain outline. The overlay (in green) marks the area that has been automatically delimited as the pollen grain area. Red arrows indicate errors that originate from detritus or other pollen grains near the target pollen grain.

Our approach is based on the automatic detection of birch pollen grains in pollen samples and the automatic detection of the pollen grain outline for size measurements. The approach includes three major steps:

1. Pollen recognition: In the first step, the TOFSI algorithm (Theuerkauf et al., 2023) is used to detect and classify pollen grains in scanned pollen samples from lake sediments and peat. TOFSI consists of two convolutional neural networks. The ‘detector’ detects pollen grains in the sample matrix, while the ‘classifier’ classifies the detected objects. We use recognition models trained to classify ~20 pollen and spore types, including birch pollen. Only the latter are relevant to the present study.
2. Selection of pollen grains suitable for measurement: Birch pollen grains identified in the first steps may occur in different orientations and states of preservation. For size measurements, only intact grains in polar view are suitable. Therefore, we implemented and trained a Resnet‐18 neural network (He et al., 2015) to distinguish ‘suitable’ from ‘unsuitable’ birch pollen grains. We trained the model, which was pre‐trained with standard image libraries, using birch pollen images from samples mounted in silicone oil and glycerol so that the model is able to recognise birch pollen of different appearance. The training data set contains ~500 birch pollen grains suitable for size measurements and 500 birch pollen grains unsuitable for size measurements (Figure 2).
3. Outline detection: To measure pollen grain sizes, we determine the outline of each pollen grain using a semantic segmentation approach, employing a DeepLab V3 neural network with a ResNet‐50 backbone (Chen et al., 2017). This network was pre‐trained on the COCO dataset (Lin et al., 2014) and further trained on a dataset of ~200 birch pollen grain images with corresponding masks (Figure 3).

### 2.2 Images

All images were taken using a modified Zeiss Axioskop 40 microscope, equipped with a motorised Prior Optiscan XY‐stage and a motorised Prior focus drive. Images were captured using a Jenoptic Gryphax Subra microscope camera mounted on a Zeiss 60 C 2/3″ 0.63× video adapter. The image resolution is 1920 × 1200 pixels. Images were acquired with ~10% overlap. To account for the low depth of field, at each position 13 images were taken at different focus levels. Each set of 13 images was stored as a Tiff image stack, with jpeg compression applied. These image stacks were used in the first step of our approach, i.e., pollen recognition with TOFSI. In subsequent steps, only the image layer with the highest contrast was used.

### 2.3 Model application

**FIGURE 4:**
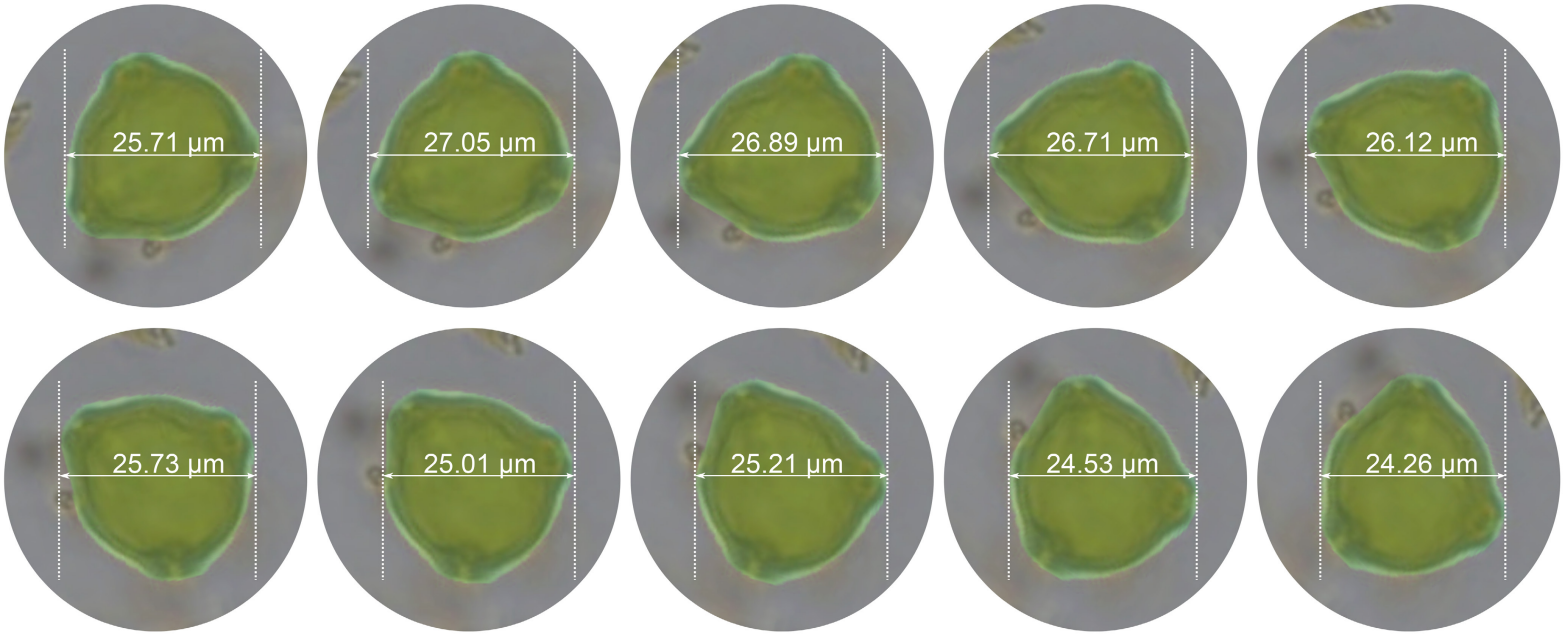
Illustration of the measured width in our approach. For each grain, the total width of each grain was measured 180 times, turning the image by 1 degree after each step. The resulting mean width is somewhat larger than the traditionally measured grain diameter from one pore tip towards the opposite wall (cf. Table 2).

All the resulting stacked images of each sample were first analysed with TOFSI for automatic pollen recognition. From all the birch pollen found in each sample, all those suitable for measurement were then selected using the RESNET‐18 neural network. Finally, the contours of all selected grains were determined using the DeepLab V3 neural network. From the resulting pollen grain outlines, two size parameters were determined: the area (μm^2^) and the mean width (μm) of each pollen grain. Areas do not refer to the actual curved surface area of the pollen grains but to the area occupied in a polar view image of the pollen grains. Hence, the area of each grain is simply the number of image pixels within each grain outline multiplied by the calibrated pixel area. Because many birch pollen grains are not symmetrical, we could not implement an automatic measurement of the grain diameter in the way that diameters have been estimated previously, i.e., as the distance from the tip of a pore to the opposite outer wall margin. Instead, we determine the mean grain width. In contrast to the diameter, width is the maximum horizontal extension of a pollen grain (Figure 4). To estimate the mean width, we rotate each image 180° in 1° steps and measure the width after each step. The mean diameter is the mean of the 180 measurements. The mean width is about 1 μm larger than the traditional grain diameter in our reference material (Table 2). All steps of the image recognition and measurement have been implemented in Python.

### 2.4 Example data

We illustrate our approach using modern reference material and pollen samples from two palaeolake records, i.e., Kieshofer Moor and Lichtenberg (Table 1). The modern reference material was collected from flowering birch specimens at various locations in northern Germany and Poland (Figure 1). These samples were treated only with acetolysis and mounted in silicone oil (Table 1).

**TABLE 1:** Origin and sample preparation of pollen samples used in present study.

Data set/location	Period	Sample preparation and mounting
Modern samples of *Betula nana*, *B. pendula*, *B. humilis* and *B. pubescens* pollen were collected in Northern Germany (Greifswald, Kiel) and Poland (Figure 1).	Modern	Sample treatment: Acetolysis (7 min at 100°C), Mounting in silicone oil
Core KM23B from the centre of palaeolake ‘Kieshofer Moor’ near Greifswald, north‐eastern Germany (Figure 1). Complete pollen analysis at 1 cm resolution ongoing.	Lateglacial to mid Holocene (~13.000–7000 cal. BP)	Sample volume: 0.8 cm^3^ Sample treatment: Addition of 2–3 Lycopodium spore tablets (Lund University) as the exotic marker (Stockmarr, 1971), 10% HCl for 10 min at 100°C 10% KOH for 10 min at 100°C Cold 40% HF for 4 days (only from 542 to 667 cm) 2× washing with acetic acid Acetolysis for 7 min at 100°C, Mounting in silicone oil Scanning within days/weeks after sample preparation
Core Li‐BPa from a Neandertal site at the margin of a palaeolake near Lichtenberg, eastern Lower Saxony, Germany (Hein et al., 2021, Figure 1). Pollen analysis of the ~18 m long sequence has not yet been published.	Late Saalian to Early Weichselian, including the Eemian interglacial (late MIS 6 to early MIS 4)	Sample weight: 5 g Sample treatment: Addition of 5 Lycopodium spore tablets (Lund University) as the exotic marker (Stockmarr, 1971) 10% HCl until end of reaction 10% KOH Flotation using sodium metatungstate hydrate Acetolysis for 10 min at 100°C Mounting in glycerol (for details, see e.g. Urban & Bigga, 2015) Scanning 2–3 years after sample preparation

We included two fossil datasets because the samples from each dataset were prepared and mounted differently, so birch pollen grains from the Kieshofer Moor dataset are usually smaller and darker than birch pollen from the Lüneburg dataset (Table 1). Neural networks such as TOFSI are sensitive to such image characteristics. Using the two datasets, we tested whether our approach is suitable for both types of data. In addition, the Lüneburg samples were mounted in glycerol, which usually implies some swelling of the pollen. The degree of swelling is variable, ranging from 10% to 80% in examples from Beug (2004). Furthermore, swelling appears to continue for the first 3 years after sample preparation and then largely ceases (Wei et al., 2023). We have included the Lichtenberg record to test whether, despite these size changes, birch pollen sizes change in a meaningful pattern over the glacial–interglacial transitions.

For the Kieshofer Moor record, the Lateglacial and Early Holocene sections have been analysed for continuous samples at 1 cm resolution. The Lichtenberg record has been analysed at variable, 5–10 cm resolution.

### 2.5 Evaluation

We separately tested the performance of the three steps of our approach. First, to test the performance of automatic pollen analysis with TOFSI, we randomly checked the images selected for measurement for the presence of false positives, e.g., *Corylus* or *Alnus* pollen grains wrongly identified as *Betula* (for revision, all measured images are available at https://zenodo.org/doi/10.5281/zenodo.11005399). To check for false negatives, i.e. *Betula* pollen grains that have not been detected by TOFSI, we for a selection of samples manually checked all images. Finally, we compare TOFSI counts from the KM23B record with manual pollen counts from the previous yet unpublished pollen record KM1.

To evaluate the actual size measurement, a number of test images were also measured manually. For this purpose, the outlines of the pollen grains were drawn manually.

### 2.6 Data analysis and presentation

For modern pollen from four birch species, the mean and standard deviation of the grain area and the mean width have been estimated (Table 2). For the fossil pollen samples from the Kieshofer Moor and Lichtenberg, size distributions of the area and mean width are shown as density plots created with the ‘geom_density’ function from the ‘ggplot2’ R package in version 3.4.2 (Wickham, 2016). As the number of birch pollen was low in several of the Kieshofer Moor samples, results were binned over five consecutive, 1‐cm samples. Particularly in very crowded samples, the final step of our approach, outline detection, may in some cases, perform incorrect (Figure 3). To filter out exceptionally small and large objects, we removed outliers with the z‐score method, i.e., measurements with a z‐score larger than 2.5. This step was applied twice.

Moreover, we tentatively try to estimate the proportion of pollen from individual birch species. To this end, we implemented an approach similar to the ‘Approximation through theoretical compound distribution approach’ from Usinger (1978) in the R environment for statistical computing (R Core Team, 2023). This approach derives from the assumption that for each birch species, the size of pollen follows an unimodal, normal distribution. In a mixed sample, i.e., a fossil or artificial sample with pollen from two or more birch species, the resulting grain size distribution is instead different, either skewed or even bi‐ to multimodal. The approximation approach tries to find, for a given sample size, which birch pollen spectrum gives a size distribution that is most similar to the observed size distribution. To find that spectrum, we apply optimization with the ‘DEoptim()’ function from the ‘DEoptim’ R package (Mullen et al., 2011). Currently, the approach can be applied to samples in which pollen of up to four birch species are expected, further can be added. As an input parameter, the calculations require the mean and the standard deviation of the pollen grain size of each of the birch species expected in a given record. We have automatically measured these parameters yet only for samples mounted in silicone oil (Table 2). We, therefore, apply the approximation approach only for the samples of the Kieshofer Moor record that are also mounted in silicone oil.

**TABLE 2:** Automatically estimated mean width and area of modern birch pollen from four birch species, all mounted in silicone oil.

Taxon	Mean width (mean/SD)	Area (mean/SD)	Diameter manual (50 pollen, mean/SD)	Birks (1968) Silicone oil	Mäkelä (1996) Silicone oil	Caseldine (2001) Silicone oil	Beug (2004) Glycerol	Blackmore et al. (2003) Glycerol
*B. nana*	20.54/0.92 (*n* = 107)	311.7/26.4 μm^2^ (*n* = 107)	18.99/1.08	18.60–18.72	17.31	19.23	21.8–(24.5)–27.0	21.0–(24.0)–27.0
*B. humilis*	22.14/0.99 (*n* = 104)	359.9/33.9 μm^2^ (*n* = 104)	21.10/1.11				22.0–(25.1)–29.5	21.0–(24.5)–27.0
*B. pendula*	22.41/0.88 (*n* = 339)	372.0/29 μm^2^ (*n* = 339)	21.28/0.96		21.37		25.5–(28.9)–31.8	22.0–(24.5)–28.0
*B. pubescens*	25.46/1.11 (*n* = 66)	478.6/39.7 μm^2^ (*n* = 66)	24.28/0.96	22.63–23.31	25.19	24.06–26.94	28.8–(30.8)–33.0	23.0–(27.0)–29.0
*B. tortuosa*				23.43–26.42				28.0–(30.0)–32.0

*Note*: For comparison, manually measured diameters from previous studies (with pollen mounted in silicone oil and glycerol). All numbers in μm, except grain areas.

## 3 RESULTS AND INTERPRETATION

### 3.1 Model test and reference data

**FIGURE 5:**
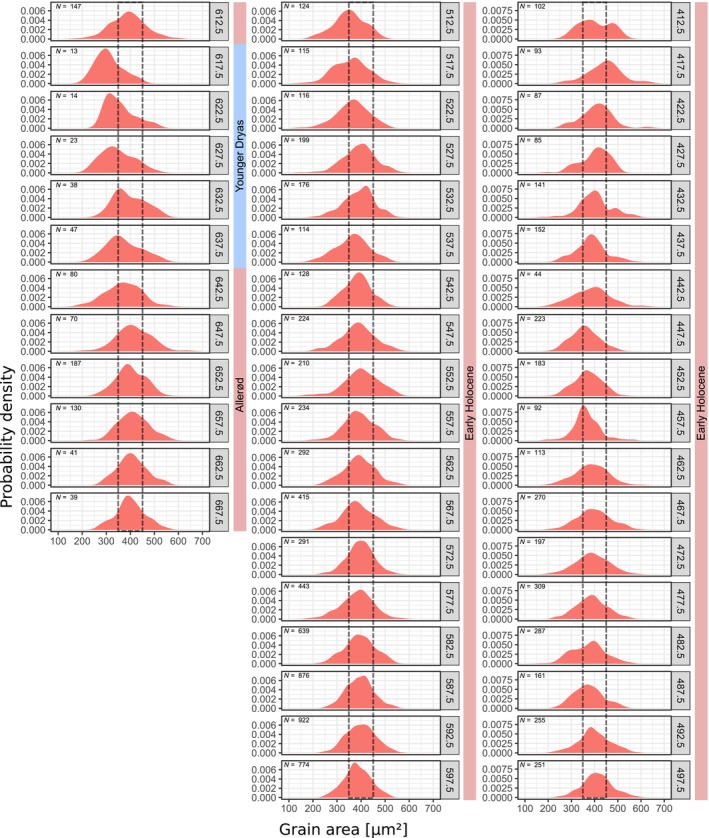
Size distribution of the automatically measured area of birch pollen in the KM23B record. Samples from 410 cm to 670 cm measured and binned in 5 cm intervals. Grain size interval 350–450 μm^2^ indicated for orientation.

For KM23B, comparison with manual pollen counts and manual revision of four samples (see Figure 5) confirms that TOFSI detected almost all *Betula* pollen grains and that almost all grains classified as *Betula* were, in fact, *Betula* pollen. Revision of the pollen measured for KM23B and Li‐BPa shows that only in very rare cases (less than 1 in 1000), misclassified pollen grains were measured (see Supporting information).

**FIGURE 6:**
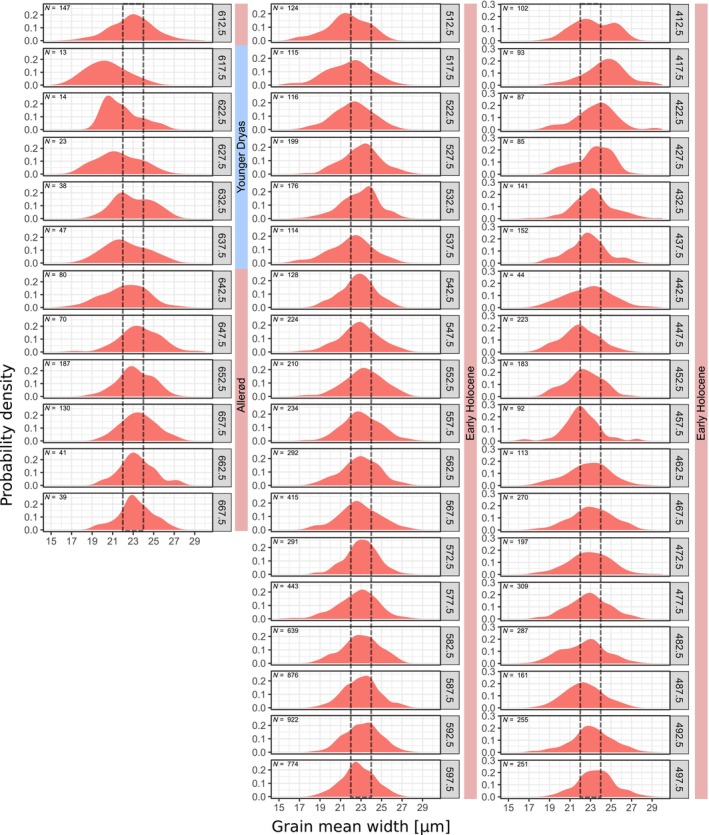
Size distribution of the automatically measured mean width of birch pollen in the KM23B record. Samples from 410 cm to 670 cm measured and binned in 5 cm intervals. Interval 22–24 μm^2^ indicated for orientation.

Manual revision confirms that the automatically determined outlines are correct in most cases. Only for a small number of images, for example, such with several overlapping pollen grains and such with much dirt, outlines are incorrect (Figure 3). In the Kieshofer Moor samples, which are rich in detritus, such errors occur in ~1.5 of 100 birch pollen grains. Such errors are virtually absent in the cleaner samples from the Lichtenberg record. The size of manually and automatically determined outlines is largely similar (Pearson correlation test: *r* ^2^ > 0.95, *p*‐value <2.2e‐16), with automatic measurements in rare cases being up to 5% larger. In those cases, the automatically determined outline usually does not capture well the concave shape near the pore. We consider this difference as negligible.

The automatically determined mean width is, for all species, about 1–1.5 μm larger than manually measured diameters, hence a direct comparison of both parameters is not useful. As in previous studies, manually and automatically measured grain sizes increase in the order of *B. nana*, *B. humilis*, *B. pendula* and *B. pubescens* (Table 2). Our manually measured diameters for *B. nana* are similar to those of Birks (1968) and Caseldine (2001), yet larger than those of Mäkelä (1996). For *B. pendula*, our manual estimates are similar to those of Mäkelä (1996). For *B. pubescens*, the manual estimates fit well into the larger range of previous estimates. We hence conclude that the presented estimates from automatic measurements, which are not directly comparable with previous estimates, provide a suitable baseline for future automated measurements.

### 3.2 Kieshofer Moor (KM23B)

**FIGURE 7:**
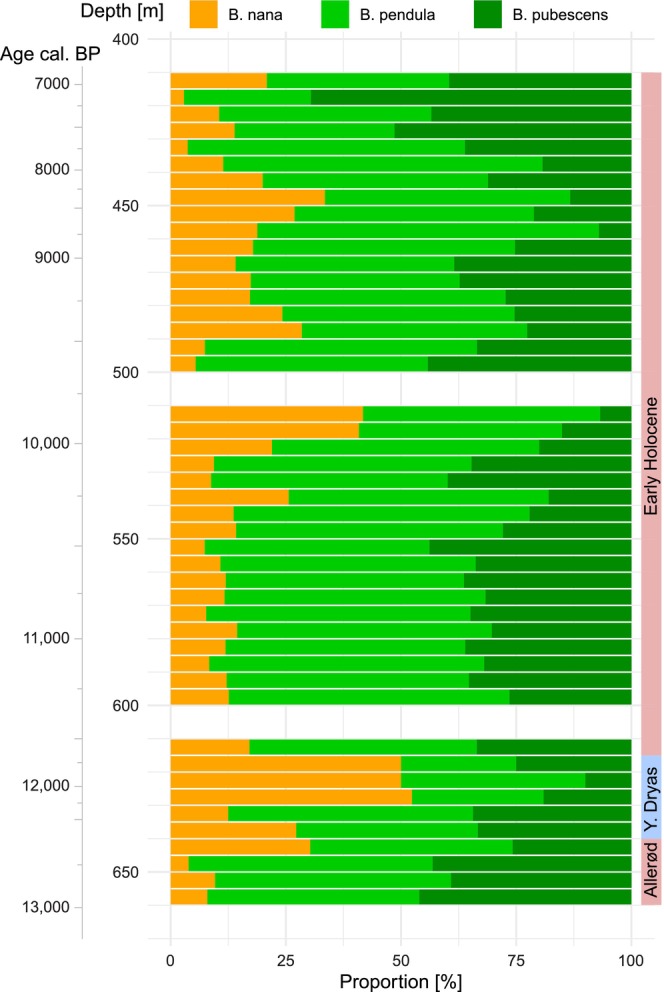
The tentative proportion of pollen of *B*. nana, *B*. pendula and *B*. pubescens in the KM23B record as estimated with the approximation approach (cf. Usinger, 1978). *B*. humilis was excluded from the analysis because this species was likely not present in the study region during that period.

The analysed core section comprises lake sediments from the Weichselian Lateglacial (660–615 cm) and the early to mid‐Holocene (615–400 cm) (Figures 5, 6, 7). According to the pollen stratigraphy, the Lateglacial section can be separated into an Allerød part (660–640 cm) and a Younger Dryas part (640–615 cm) with a markedly increased proportion of herbal pollen types (Theuerkauf et al., 2014). The position of the Allerød is confirmed by the presence of the Laacher See Tephra at 654 cm. The KM23B core has been sampled and continuously analysed at 1 cm resolution. As in several samples the number of measured birch pollen grains is below 25, we pooled samples in 5 cm intervals.

In the Allerød section (660–640 cm), the grain size distribution appears unimodal, with a mean area of ~400 μm^2^ and a mean width of ~23 μm. With the onset of the Younger Dryas at 640 cm, grain sizes begin to decline until in section 615–620 cm, smaller grains with an area of ~300 μm^2^ and a mean width of 20 μm dominate. After the onset of the Holocene at 615 cm, grain sizes immediately increase again to values similar to those in the previous Allerød section (area ~ 400 μm^2^/mean width ~ 23 μm). Grains of that size class dominate also in most samples of the following early Holocene sequence, with some exceptions. Smaller grains are again more abundant at 510–520 cm, 480–485 cm and 440–460 cm. Larger grains are present in the topmost section at 410–420 cm, as indicated by a peak in the grain size distribution at 475 μm^2^/25.5 μm.

Overall, it appears that in the KM23B record, at least three size classes of birch pollen are present, with mean sizes of around 300 μm^2^/20 μm, 400 μm^2^/23 μm and 475 μm^2^/25.5 μm. Comparison with our reference material suggests that these classes correspond to *Betula nana*, *B. pendula* and *B. pubescens*. We applied the approximation approach under the assumption that only these three taxa were present in the study region. The results suggest that during the Allerød period, about 90% of the birch pollen is from tree birches, with equal amounts from *B. pendula* and *B. pubescens* (Figure 7). During the following Younger Dryas cold period, the proportion of pollen attributed to *B. nana* increases to above 50%. After the onset of the Holocene, tree birch pollen was again dominant, with ~55% being interpreted as *B. pendula* pollen and ~ 35% being interpreted as *B. pubescens* pollen. As mentioned above, the proportion of smaller birch pollen interpreted as from *B. nana* was again elevated during three early Holocene sections, to ~45% at 510–520 m, to ~25% at 480–490 cm, and to ~35% a 450 cm depth. In these sections, mainly the proportion of pollen interpreted as from *B. pubescens* is reduced. The proportion of pollen interpreted as from *B. pendula* is instead stable. Only in the top sections, namely, at 415–420 cm, the proportion of pollen interpreted as from *B. pubescens* is clearly higher.

### 3.3 Lichtenberg (Li‐BPa)

**FIGURE 8:**
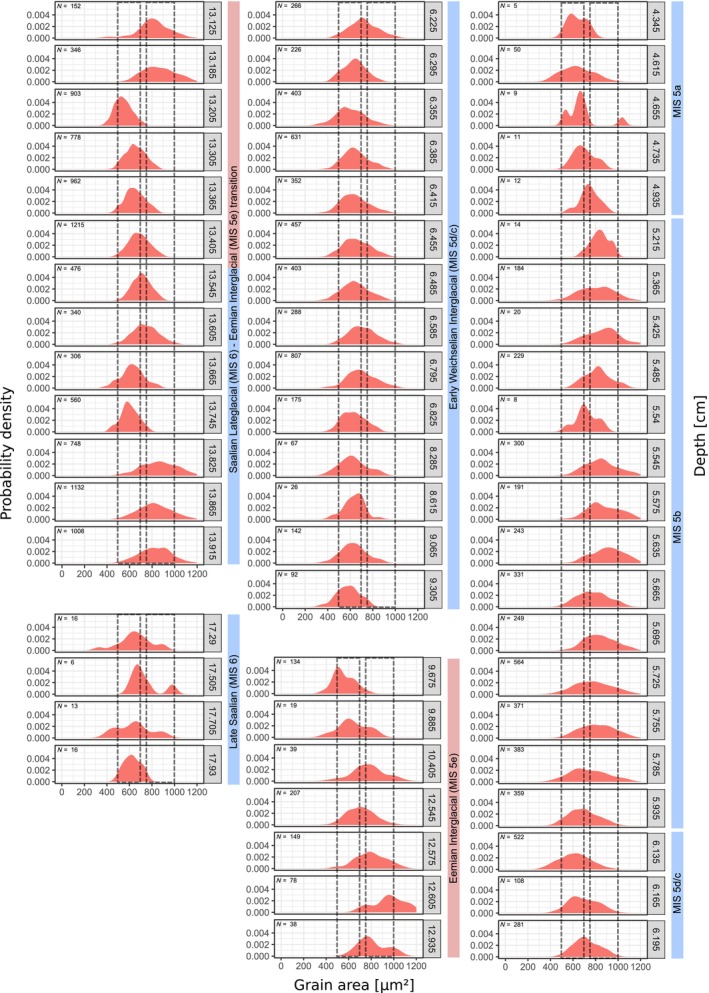
Size distribution of the automatically measured birch pollen area in the Li‐BPa record. Grain size intervals 500–700 μm^2^ and 750–1000 μm^2^ indicated for orientation. For measurements of the mean width, see Supporting information.

**FIGURE 9:**
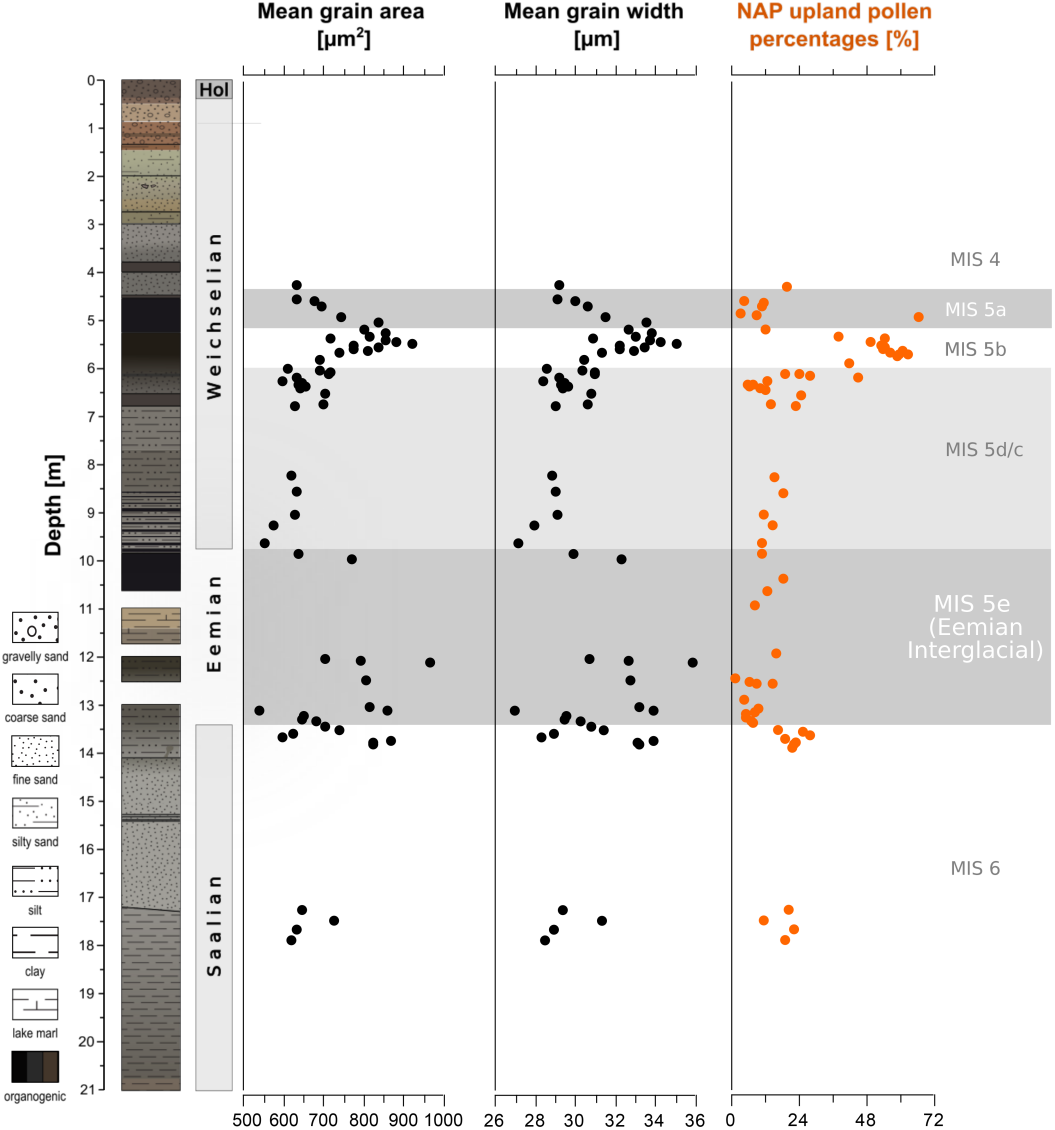
Sediment stratigraphy (left), Betula grain sizes (as grain area and mean grain width) and proportion of NAP upland pollen in the Li‐Bpa record (Hein et al., 2021). NAP upland pollen percentages are derived from automatic pollen counts with TOFSI (Theuerkauf et al., 2023). The zonation of the sequence is based on a conventionally established, yet unpublished pollen record (data by Brigitte Urban).

In the Li‐BPa record, we mainly analysed samples from pollen‐rich sections with sufficient numbers of birch pollen, including samples from the Late Saalian Warthe stage (~17.2–14.1 m), correlated with the Marine Isotope Stage MIS 6 (Lisiecki & Raymo, 2005), from the transition of the Saalian Lateglacial into the Eemian Interglacial (~14.1–13.5 m), from the Eemian Interglacial period (~13.5–9.75 m) (MIS 5e) and from the Early Weichselian Glacial period (~9.75–4.0 m) (MIS 5a–d and MIS 4) (Hein et al., 2021). Pollen‐stratigraphically, only the Rederstall Stadial (MIS 5b) and the Odderade Interstadial (MIS 5a) are detectable within this section (Figures 8 and 9).

Birch pollen grains from the Li‐BPa record are significantly larger than those from KM23B. We suggest that the larger size is mainly due to the fact that these samples were mounted in glycerol rather than silicone oil, but will also discuss alternative reasons later. The grain area ranges from ~400 to more than 1000 μm^2^, the mean width ranges from ~23 to 40 μm. Along the record, we observe several prominent changes in grain size.

Birch pollen from the Late Saalian stage (18–17 m) mostly have an area of 600–700 μm^2^ and a width of 27–32 μm (Figures 8, 9). Around the transition from the Saalian Lateglacial to the Eemian Interglacial (14–13 m) grain sizes vary considerably. Birch pollen grains are large in the lower part of this section (750–1000 μm^2^/32–37 μm at 13.78 to 13.87 m), smaller in the middle part (500–800 μm^2^/25–33 μm at 13.16 to 13.70 m), and again larger in the uppermost samples of this section (700–1000 μm^2^/30–37 μm at 13.08 to 13.14 m). In the samples from the Eemian Interglacial itself, birch pollen size is mostly around 600–900 μm^2^/29–35 μm, with a substantial decrease towards the end of the interglacial.

During the first part of the Early Weichselian (~9.50–6.50 m), birch pollen grains are again mostly small (500–700 μm^2^/27–31 μm). Grain sizes then increase above ~6.50 m, to maximum values of 700–1000 μm^2^/32–37 μm at about 5.50 m. From 5.50 to 4.50 m, grain sizes again decline to ~500–700 μm^2^/27–31 μm.

Interpretation of the Li‐BPa results in terms of tree birch species is hampered because we lack measurements from modern reference material that has been prepared similarly. The size distributions do not recognisably cluster around particular mean values, which may represent a single species. Rather narrow, unimodal distributions, which may represent a single species, show very different mean values, for example, 500 μm^2^ (13.2 μm), 600 μm^2^ (12.54 m, 9.06 m), 700 μm^2^ (13.54 m), 800 μm^2^ (12.57 m, 5.48 m, 5.54 m) and finally at 1000 μm^2^ (at 5.57 m, 13.82 m). Our preliminary interpretation is that the smaller grains (~500–700 μm^2^/~27–31 μm) are attributable to dwarf birches, while larger grains (750–1000 μm^2^/32–35 μm) are attributable to tree birches. Hence, the Li‐BPa record indicates several changes in dwarf versus tree birch presence, which largely correspond to major climatic variations. During the cold Late Saalian stage mainly dwarf birch was present. At the transition from the Saalian Lateglacial to the Eemian interglacial, our results indicate a shift from dwarf to tree birch dominance, which well corresponds to the warming of the Eemian Interglacial and the subsequent expansion of forests. The expansion of tree birches appears to have been interrupted, as again smaller birch pollen dominate from 13.70 to 13.20 m. One explanation of this pattern would be a cold event as described, for example, in Beets et al. (2006), Helmens (2014) and Strahl (2016), causing a decline in tree birch and a return to again more open vegetation. As expected, our results indicate that tree birch prevailed during most of the following Eemian Interglacial, only declining towards the end of the interglacial in favour of dwarf birch. Smaller birch pollen then indicates the presence of mostly dwarf birch since the onset of the Early Weichselian. Again, larger grain sizes at 5.50 m may suggests another expansion of tree birch in the vicinity of the Li‐BPa site. We discuss the possible meaning of this event in the following chapter.

## 4 DISCUSSION

The two example records show that our machine learning approach is well‐suited to carry out fast automatic size measurements of birch pollen grains, and potentially other microscopic objects. Given a sufficient number of pollen grains is present in the samples, several thousand birch pollen grains can be measured per hour. The measurements are standardised, i.e., well‐comparable between samples and potentially between labs. The necessary images have been taken using a standard light microscope, equipped with a motorised stage and focus drive. A minor drawback is that the two size parameters estimated in our approach, grain area and mean width, differ from parameters measured previously, usually the grain diameter from the tip of a pore to the opposite wall. We consider this limitation to be minor because measurements of modern reference material can well be conducted rapidly using the approach presented.

Errors in the automatic pollen recognition and size measurements are small overall. A comparison of manual and automatic pollen counts from the Kieshofer Moor shows that KM23B pollen values are reliable. Furthermore, manual revision of four samples shows high recall and precision, i.e., almost all birch pollen grains were detected and almost all pollen grains classified as *Betula* were in fact *Betula* pollen (see Supporting information). Only in rare cases was *Betula* pollen mistaken for *Alnus* or *Corylus* pollen, so that indeed, with very few exceptions, only *Betula* pollen grains were measured. Pollen suitable for measurement was also reliably selected, with errors usually less than 5%. Finally, the measurements themselves are also reliable. In the Kieshofer Moor samples, the majority of birch pollen could not be measured due to their unfavourable orientation. We do not assume, but cannot exclude, that this may have biased the results.

The two example records from Kieshofer Moor and Lichtenberg underline the possibilities of the approach, and also highlight the current difficulties in the interpretation of birch pollen size measurements. In the Kieshofer Moor record, we observe predominantly larger, probably tree birch pollen during the Allerød warm period, a shift towards smaller, probably dwarf birch pollen during the cold Younger Dryas and again predominantly larger, probably tree birch pollen after the onset of the Holocene. These changes agree well with earlier observations, which show a dense forest cover during the Allerød, largely open vegetation during the Younger Dryas, and again forest vegetation during the early Holocene (de Klerk, 2008, Theuerkauf et al., 2014; Theuerkauf & Joosten, 2009, 2012). It appears that the decline of tree birch after the onset of the Younger Dryas was rather gradual, whereas the expansion of tree birch after the onset of the Holocene was very rapid. Whether this pattern is peculiar to the Kieshofer Moor area or common to northern Central Europe needs further validation.

Application of the approximation approach suggests that two tree birch species, *B. pendula* and *B. pubescens*, were well present since the onset of the record around 13,000 years ago. For the neighbouring area of Schleswig‐Holstein/Northern Germany, pollen and macrofossils instead suggest that *B. pubescens* was the dominant tree birch during the Lateglacial, whereas *B. pendula* was present only after the beginning of the Holocene (Usinger, 1981). Macrofossils of *B. pendula*, like those of *B. pubescens*, are present in the Paddenluch near Berlin/Eastern Germany since at least 14,000 years ago (Kossler, 2010). The present result may indicate that *B. pendula* was mainly present in the eastern, more continental parts of Germany during the Late Glacial. However, further analysis is needed to prove such a pattern.

An unexpected result of the Kieshofer Moor record is the again increased proportion of pollen interpreted as from *B. nana* during the Early Holocene, at ~9800–10.000 cal. BP, ~9300–9400 cal. BP and ~8100–8800 cal. BP. At the moment, we refrain from interpreting this finding as some regional scale expansion of dwarf birch following widespread forest decline, for example, in response to a pronounced cold event such as the 8.2 ka event (Rasmussen et al., 2008). Such cold events should have first affected the more warm‐loving tree taxa, such as *Quercus*, *Ulmus* and *Tilia*, but this is not evident in KM23B or other pollen records from the region (Theuerkauf et al., 2014). Instead, the early Holocene peaks in *B. nana* pollen may reflect local stands of dwarf birch along the margins or in the vicinity of the palaeolake. Small local stands may have survived since the Younger Dryas. Their existence is more difficult to demonstrate for the first ~1600 years of the Holocene when overall birch pollen deposition was high due to widespread birch forests. In the Kieshofer Moor, birch pollen deposition peaked at 30,000 grains cm^−2^ yr^−1^ at around 11,500 cal. BP (Theuerkauf et al., 2014). The birch forest cover declined after 10,000 cal. BP due to the expansion of thermophilic deciduous tree taxa and birch pollen deposition dropped to well below 10,000 grains cm^−2^ yr^−1^. Now, the rarer dwarf birch pollen could be again a visible part of the total birch pollen deposition. For a final interpretation of the present findings as a local or regional effect, validation from further sites, including macrofossil evidence, is needed.

Interpreting the size measurements of the Li‐BPa record is more difficult for two main reasons. First, the samples were mounted in glycerol, which causes pollen grains to swell to a variable degree (Andersen, 1960; Beug, 2004). Birch pollen from Li‐BPa is correspondingly much larger than those mounted in silicone oil from KM23B (Table 3). They are also larger than the measurements of birch pollen mounted in glycerol jelly by Blackmore et al. (2003) and Beug (2004) (Table 2). We assume that the larger size is related to the fact that we scanned and measured Li‐BPa birch pollen only 2–3 years after sample preparation. According to Wei et al. (2023), the swelling of Poaceae pollen in glycerol jelly continues for about 3 years and then slows down. However, we do not know whether Blackmore et al. (2003) and Beug (2004) measured fresher samples.

**TABLE 3:** The mean size of assumed dwarf and tree birch pollen in the KM23B and Li‐BPa records.

	dwarf birch pollen	Tree birch pollen
KM23B	300 μm^2^/20 μm	400 μm^2^/23 μm
Li‐BPa	600 μm^2^/29 μm	850 μm^2^/34 μm

Pollen grain size is also influenced by sample preparation, namely, acetolysis (e.g. Andersen, 1960; Reitsma, 1969). The preparation of our samples differed mainly in the duration of acetolysis, which was 10 min for Li‐BPa and 7 min for KM23B. According to Reitsma (1969), acetolysis causes an initial swelling of the pollen grains, followed by a slow shrinkage. The effect on grain size should be stable after 4 min. The longer acetolysis for Li‐BPa samples is, therefore, unlikely to explain the larger birch grain sizes in this dataset.

The second difficulty is the complex stratigraphy of the Li‐BPa sediment record, with its numerous sedimentological changes and hiatuses also within the Eemian Interglacial and the Early Weichselian sequence. We nevertheless observe size changes in birch pollen that follow the major climatic variations. Birch pollen grains are mostly larger during the Eemian Interglacial than during the Saalian Pleniglacial and the Early Weichselian Glacial, reflecting the well‐known pattern of open vegetation with predominant dwarf birch during the glacial periods and forest vegetation with predominant tree birch during the Eemian Interglacial. While these long‐term trends follow the expected pattern, the interpretation of two short‐term peaks in birch pollen sizes remains more difficult. The first peak includes the three samples from 13.82 to 13.92 m with large, probably tree birch pollen. Pollen‐stratigraphically, this section is located slightly below the onset of the Eemian interglacial (MIS 5e), which is identified by a prominent decline in NAP values at ~13.50 m. Birch pollen sizes increase from about 13.80 to 13.20 m, well corresponding with the transition from open to forest vegetation indicated by the decreasing NAP upland values. The samples with probable tree birch pollen from 13.82 to 13.92 m are still characterised by high NAP upland values, indicating a rather open vegetation. This observation may hence point to an early expansion of some birch trees, probably in the vicinity of the coring site, in an otherwise still open landscape. This expansion was then obviously again interrupted, possibly by a cold event. A cold‐dry event at the Saalian‐Eemian boundary has been described by e.g. Beets et al. (2006). We consider this interpretation as tentative because the Saalian Lateglacial is only partly present in the Li‐BPa record, as indicated by the sedimentary boundary just below at 14.10 m (Figure 9). We, therefore, cannot evaluate the complete expansion pattern of tree birch at the Saalian–Eemian boundary. Also, some disturbance of the sediment section including the larger birch pollen from 13.92 to 13.82 m cannot be excluded.

The second short‐term peak in birch pollen grain sizes is at around 5.50 m. In northern Germany, at least a short tree birch phase, followed by a longer pine phase, has been described for the Odderade interstadial (MIS 5a, e.g., Behre & Lade, 1986, Behre et al., 2005). The samples with larger birch pollen at ~5.50 m are still characterised by high percentages of non‐arboreal pollen, including pollen from heliophile taxa like grasses and *Artemisia*, which still point at stadial conditions. The peak in birch pollen sizes may indicate a very first expansion of some patches of tree birch in an otherwise still open vegetation, prior or during the onset of the Odderade Interstadial or is more likely due to sediment disturbances or mixing around the beginning of the interstadial deposition.

As mentioned above, variations in the size of birch pollen have also been related to technical issues, i.e., the sample preparation and the time since preparation. We assume that these factors play a minor role in the Li‐BPa record because all samples have been prepared using the same protocol and have been scanned at about the same time after sample preparation, about 2–3 years. Andersen (1960) also mentions unexplained cases of samples with very large birch pollen. As the two peaks above include several samples with larger birch pollen, we assume that such random effects do not apply here. It shall be noted that the size peak at 5.50 m falls into a sediment section with high organic content. The larger grains size may hence be related to taphonomic effects caused by sediment properties. Praglowski (1966) indeed has discussed some influence of, e.g., peat degradation on the size of various pollen types, yet the magnitude and underlying mechanism of such effect are yet poorly understood. In summary, the two birch pollen peaks may reflect very early periods of tree birch expansion in still widely open landscapes. However, this observation needs validation from further, more continuous records, and with samples being analysed immediately after preparation.

### 4.1 Future steps

The present results support that automatic size measurements are indeed well‐suited to distinguish periods with dominating dwarf versus tree birch pollen in fossil pollen samples. More refined results and the additional separation of different (tree) birch species may be possible as well, yet robust results still require a better understanding of the variability of birch pollen sizes and the factors influencing them. The measured pollen size may be influenced by, for example, the pollen sampling strategy, sample storage before preparation, sample preparation, the mounting medium, slide thickness and the measuring approach (e.g., Clegg et al., 2005; Mäkelä, 1996; Reitsma, 1969). Moreover, Eneroth (1951) suggests that the size of birch pollen is also influenced by climatic factors. The size of pollen from *B. nana*, *B. pendula* and *B. pubescens* tends to be 5–10% higher in higher altitude and latitude, i.e., in cooler climates (Usinger, 1980). Finally, the hybridisation of birch species may complicate the interpretation of pollen sizes (Ives, 1977).

Hitherto published measurements of modern pollen are partly inconsistent, likely due to the reasons mentioned above (Table 2). The main limiting factor for the study of birch pollen sizes so far are the time‐consuming, manual size measurements. The present automatic approach reduces those efforts substantially, as all steps of the approach run automatically and in a short time. When using a standard PC equipped with middle‐class GPU, full analysis of all images of a 15 × 15 mm sample area with potentially several thousand birch pollen takes about 1 hour. We therefore hope that our approach will trigger further systemic research on modern birch pollen, including research on the impact of environmental factors and sample preparation on birch pollen. All utilised tools, training data and instructions are freely available from the mentioned sources. We assume that because imaging devices and sample preparation procedures differ among labs, the neural networks may require additional training to adjust for the specific image characteristics. From our experience, suitable training data can be produced in a reasonable time, particularly from pollen‐rich modern or fossil material.

The performance of the trained neural networks is not yet perfect. In about 5% of cases, our selection network (step 2) did not distinguish correctly between those birch pollen grains that were suitable for measurement and those that were not. Although these errors have minimal impact on the final results, we will investigate whether the errors can be reduced with more training data or whether adjustments to the underlying neural networks will be necessary. In addition, the estimated pollen grain outlines are often slightly too large due to a small ‘halo’. We will try to reduce this effect also either with further training data or with adjustments to the underlying neural network.

Despite the problem of swelling, we found that pollen mounted in glycerol tended to settle more easily in the polar orientation suitable for measurement. Birch pollen mounted in silicone oil (we use oil with high viscosity of 2000 cSt), on the other hand, often remains in an unfavourable orientation. For this reason a majority of the birch pollen in the Kieshofer Moor record could not be measured, which may add bias in the results. This disadvantage may be removed by using silicone oil with lower viscosity. On the other hand to reduce the disturbing effects of swelling in glycerol samples, they should be scanned immediately after sample preparation.

The performance of the approximation approach to quantify the abundance of birch pollen from different taxa depends on several parameters, including the number of pollen measured and the mean and standard deviation of pollen sizes from the relevant birch species. Further tests, both with simulated and real data, are needed to explore the robustness of the approximation approach.

Several studies have shown that pore depth is a useful additional parameter to distinguish between different birch species (e.g., Birks, 1968; Clegg et al., 2005). In the next step, we will investigate whether pore depth can also be measured automatically.

Finally, automated size measurements are potentially useful for the analysis of other pollen and non‐pollen objects. Size measurements of wild grass pollen may be useful for distinguishing different grassland types in the past (Jan et al., 2015, Schüler & Behling, 2011). However, Wei et al. (2023) do not find a relationship between wild grass pollen size and taxonomic units or environmental parameters. Expanded size measurements with tools such as the one presented here may help to better understand if and in which settings wild grass pollen grain size is a useful parameter. Within our TOFSI approach to automatic pollen recognition, size measurements will be useful to distinguish between wild grass and cereal pollen. So far, further differentiation of cereal pollen, e.g., by surface ornamentation, requires manual revision with higher magnification and phase contrast. As in the present case of dwarf versus tree birch pollen, automatic size measurements will be useful to automatically distinguish other pairs of similar pollen types with a partial size difference, e.g., *Alnus glutinosa* type versus *Alnus viridis* type (sensu Blackmore et al., 2003), *Quercus robur‐pubescens* type versus *Quercus ilex* type (Beug, 2004) and finally, pollen of the two native European spruce species, *Picea abies* and *Picea omorika* (Lang et al., 2023). *Pediastrum* body size has been found to correlate with lake water temperature (Huang et al., 2023; Turner et al., 2014). Automated size measurements may therefore provide a climate proxy. Finally, microcharcoal particles are routinely counted along pollen grains. In addition to their number, their size and shape also provide valuable information. With minor adaptations, our approach will be able to detect charcoal particles and estimate their size and other morphometric parameters.

## AUTHOR CONTRIBUTIONS

**Martin Theuerkauf:** Conceptualization (lead); formal analysis (lead); methodology (equal); project administration (equal); software (equal); visualization (lead); writing – original draft (lead); writing – review and editing (equal). **Elias Nehring:** Methodology (equal); writing – review and editing (equal). **Alexander Gillert:** Methodology (equal); software (equal); writing – review and editing (equal). **Philipp Morten Bodien:** Data curation (equal); formal analysis (equal); validation (equal); writing – review and editing (equal). **Michael Hein:** Formal analysis (equal); funding acquisition (equal); investigation (equal); visualization (equal); writing – review and editing (equal). **Brigitte Urban:** Formal analysis (equal); funding acquisition (lead); investigation (equal); writing – review and editing (equal).

## CONFLICT OF INTEREST STATEMENT

The authors have no conflict of interest to report.

## Supporting information


Figures S1–S3

## Data Availability

The TOFSI tool for automatic pollen recognition (the first step of our approach) is available from https://github.com/alexander‐g/Tofsi‐POST. The algorithm to select birch pollen images that are suitable for measurement (the second step of our approach) is available from https://github.com/MartinTheuerkauf/birch‐pollen. Outline detection for the size measurement (the third step of our approach) is available from https://github.com/elnehr/birkenpollen. A Python script that combines usage of all three steps is also available from https://github.com/MartinTheuerkauf/birch‐pollen. All birch pollen grains that were automatically selected and measured for the present study are available for revision at https://zenodo.org/doi/10.5281/zenodo.11005399.
