# Supplementary material for: First automatic size measurements for the separation of dwarf birch and tree birch pollen in MIS 6 to MIS 1 records from Northern Germany
Source: Ecol Evol. 2024 Jun 14;14(6):e11510. doi: 10.1002/ece3.11510 (PMC11176728; doi:10.1002/ece3.11510)
Supplement: Supplementary file 1 — Figures S1–S3 [file ECE3-14-e11510-s001.zip › ece311510-sup-0001-FigureS1.pdf]

Pollen proportions in two pollen records from the Kieshofer Moor

Automatic counts with TOFSI from core KM23B (green)

Manual counts from core KM1 (yellow)

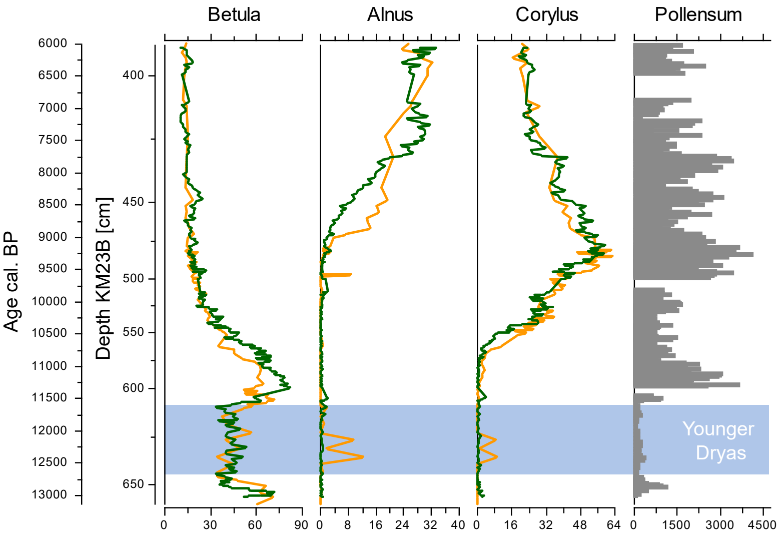

Manual revision of 4 pollen samples of the KM23B record

Recall: proportion of all pollen in the sample that has been automatically detected

Precision: proportion of automatically detected pollen that is correct

	Betula			Corylus			Alnus		
	**N**	**Recall**	**Precision**	**N**	**Recall**	**Precision**	**N**	**Recall**	**Precision**
KM23B 425-426 cm	189	0.95	0.93	349	0.94	0.99	396	0.95	0.99
KM23B 525-526 cm	330	0.91	0.99	488	0.94	0.99	15	1	0.56
KM23B 575-576 cm	592	0.96	1	12	1	1	5	1	0.75
KM23B 625-626 cm	49	0.89	0.98	4	1	0.25	0	-	-
