# Supplementary material for: First automatic size measurements for the separation of dwarf birch and tree birch pollen in MIS 6 to MIS 1 records from Northern Germany
Source: Ecol Evol. 2024 Jun 14;14(6):e11510. doi: 10.1002/ece3.11510 (PMC11176728; doi:10.1002/ece3.11510)
Supplement: Supplementary file 1 — Figures S1–S3 [file ECE3-14-e11510-s001.zip › ece311510-sup-0002-FigureS2.pdf]

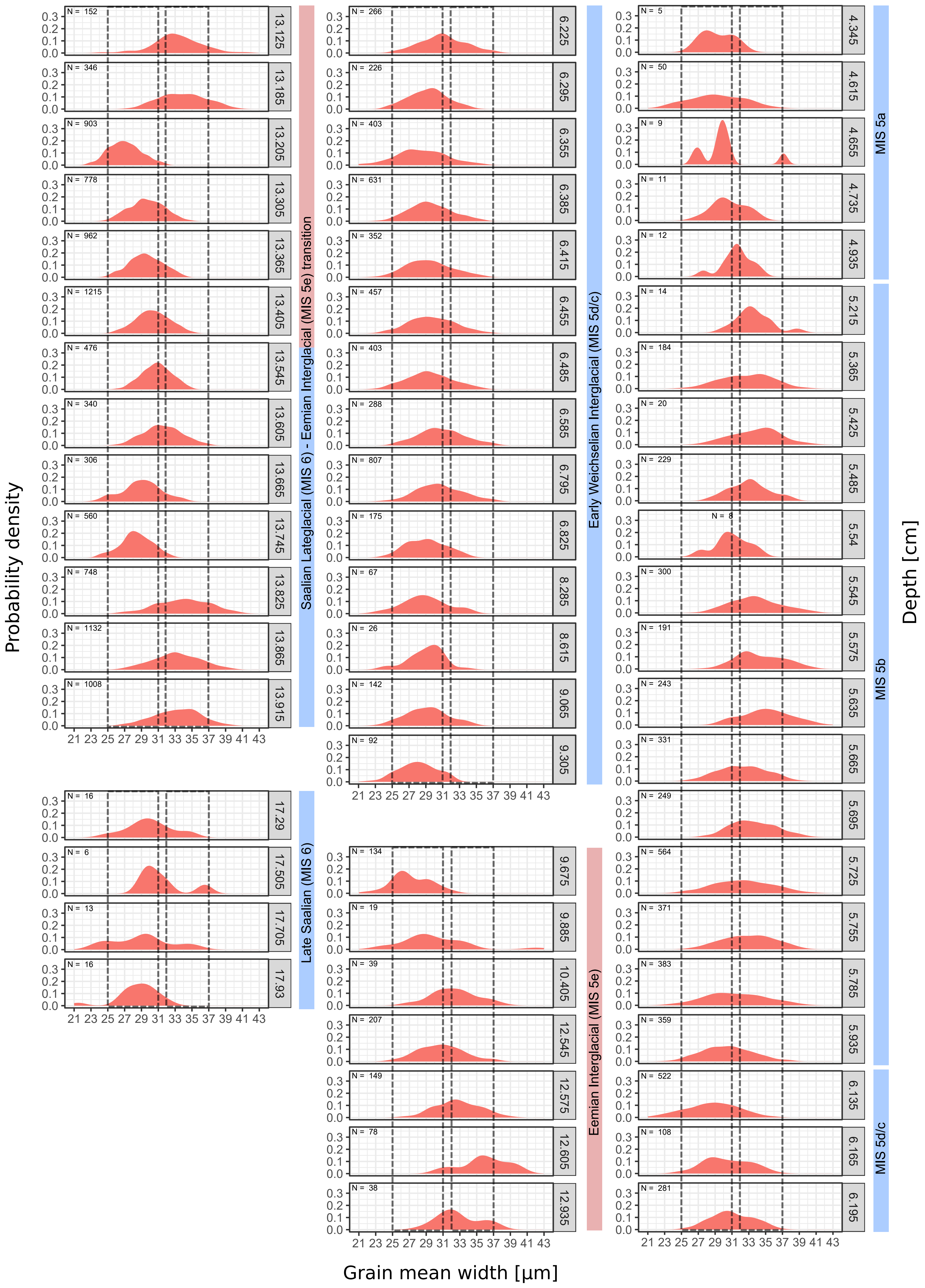
Probability density
Grain mean width [µm]
Depth [cm]
N = 152
13.125
N = 346
13.185
N = 903
13.205
N = 778
13.305
N = 962
13.365
N = 1215
13.405
N = 476
13.545
N = 340
13.605
N = 306
13.665
N = 560
13.745
N = 748
13.825
N = 1132
13.865
N = 1008
13.915
Saalian Lateglacial (MIS 6) - Eemian Interglacial (MIS 5e) transition
N = 16
17.29
N = 6
17.505
N = 13
17.705
N = 16
17.93
Late Saalian (MIS 6)
N = 266
6.225
N = 226
6.295
N = 403
6.355
N = 631
6.385
N = 352
6.415
N = 457
6.455
N = 403
6.485
N = 288
6.585
N = 807
6.795
N = 175
6.825
N = 67
8.285
N = 26
8.615
N = 142
9.065
N = 92
9.305
Early Weichselian Interglacial (MIS 5d/c)
N = 134
9.675
N = 19
9.885
N = 39
10.405
N = 207
12.545
N = 149
12.575
N = 78
12.605
N = 38
12.935
Eemian Interglacial (MIS 5e)
N = 5
4.345
N = 50
4.615
N = 9
4.655
N = 11
4.735
N = 12
4.935
MIS 5a
N = 14
5.215
N = 184
5.365
N = 20
5.425
N = 229
5.485
N = 8
5.54
N = 300
5.545
N = 191
5.575
N = 243
5.635
N = 331
5.665
N = 249
5.695
N = 564
5.725
N = 371
5.755
N = 383
5.785
N = 359
5.935
MIS 5b
N = 522
6.135
N = 108
6.165
N = 281
6.195
MIS 5d/c
0.0 0.1 0.2 0.3
21 23 25 27 29 31 33 35 37 39 41 43
