# Supplementary material for: First automatic size measurements for the separation of dwarf birch and tree birch pollen in MIS 6 to MIS 1 records from Northern Germany
Source: Ecol Evol. 2024 Jun 14;14(6):e11510. doi: 10.1002/ece3.11510 (PMC11176728; doi:10.1002/ece3.11510)

The size distribution of freshly collected pollen as determined with our algorithm.
For sample preparation, see Table 1.

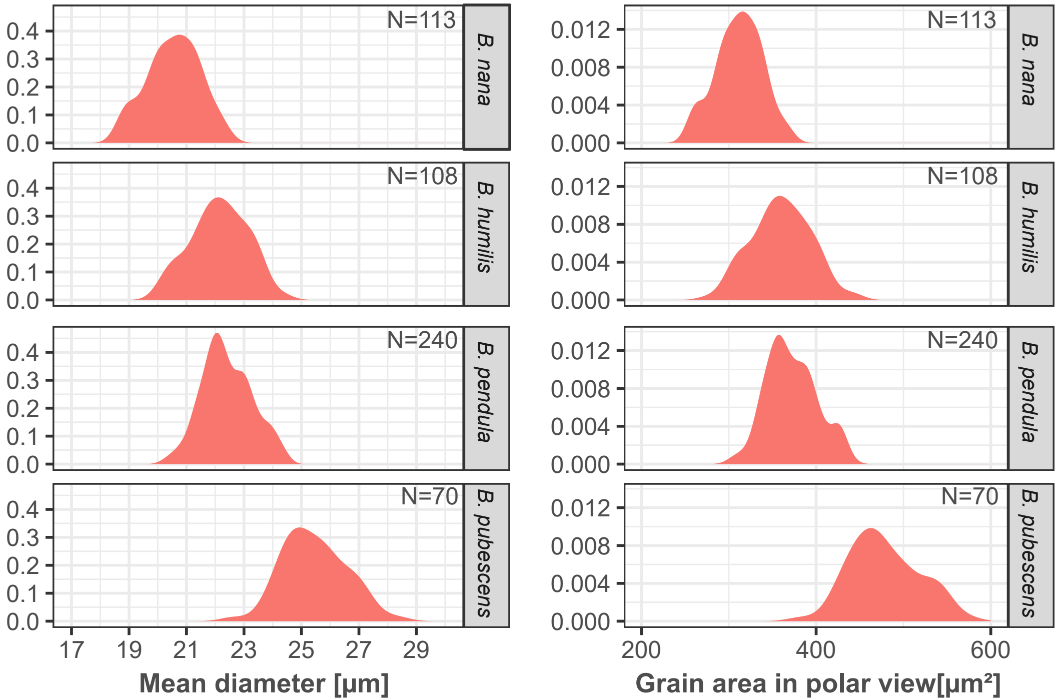

Supplement: Supplementary file 1 — Figures S1–S3 [file ECE3-14-e11510-s001.zip › ece311510-sup-0003-FigureS3.pdf]
